# A Critical Review of Children in Rural Latin America: Toward Meaningful Engagement in Complex Research Contexts

**DOI:** 10.3390/bs16050773

**Published:** 2026-05-14

**Authors:** Jazmín Mazó, Camila Jiménez-Sánchez, Gerrit Loots, Marcela Losantos

**Affiliations:** 1 Research Group BRISPO (Brussels Institute for Social and Population Studies), Department of Sociology, Vrije Universiteit Brussel, Pleinlaan 2, 1050 Brussels, Belgium; camila.jimenez@ucb.edu.bo (C.J.-S.);; 2 Instituto de Investigaciones en Ciencias del Comportamiento (IICC), Universidad Católica Boliviana “San Pablo”, La Paz, Bolivia; 3 Centro de Investigación en Ciencias Sociales (CICS), Universidad Católica Boliviana “San Pablo”, Cochabamba, Bolivia; 4 Institutional University Cooperation (IUC), 1050 Brussels, Belgium

**Keywords:** Community-Based Participatory Research, participatory action research, children, adolescent, child participation, rural communities, indigenous peoples, critical review

## Abstract

Participatory research with children has expanded globally; however, existing reviews predominantly focus on Western and urban contexts, offering limited insight into rural Latin American settings. A critical literature review was conducted to examine participatory research with children and adolescents in rural Latin America between 2018 and 2025, focusing on the reported sociodemographic characteristics, research phases and degree of children’s participation. Sixteen empirical studies met the inclusion criteria. Findings indicate that while participatory methods are widely used, decision-making authority remains concentrated in adults during early stages, with children’s involvement often limited to implementation and dissemination. The analysis reveals that the redistribution of power operates across three interrelated levels: (1) structural–methodological conditions, characterized by institutional and territorial pre-structuring and the invisibility of sociodemographic interactions; (2) relational–community dynamics, involving community hierarchies, cultural norms, and institutional actors; and (3) experiential–child engagement, where shifting roles reflect the varying degrees of agency children exercise throughout the process. Addressing these levels in an integrated manner is critical, as it is through their alignment that the redistribution of power can move beyond procedural inclusion toward more meaningful forms of participation. In this sense, meaningful participation requires a transition in which children are able to recognize themselves as active social agents and meaning-makers who can influence and shape the trajectory of research. Building on this, the study argues that such involvement depends not only on redistributing power but on employing analytical frameworks that resonate with children’s lived realities and their diverse social positions within rural territories.

## 1. Introduction

Adult perspectives have historically dominated research involving children, frequently positioning them as passive subjects rather than as active contributors to knowledge production (Biswas et al., 2024). In response to this limitation, participatory research approaches have expanded across disciplines, seeking to reduce hierarchical relationships between researchers and participants and to promote shared decision-making and collective knowledge generation (Brown, 2022; do Carmo et al., 2024; Jacquez et al., 2013; Kwan & Walsh, 2018). Within this broader field, children’s participation has increasingly been conceptualized not only as a methodological strategy but also as a developmental and civic process that fosters agency, leadership, and sociopolitical engagement (Ballonoff Suleiman et al., 2021; Kazanskaia, 2025).

To understand the participation of children, phase-based and role-based analytical frameworks have been proposed. Israel et al. (2005) describe five iterative phases of participatory research: partnership formation and maintenance, co-construction of topics, research design and implementation, joint analysis, and dissemination. Complementing this structure, models of children’s participation including Hart (1992), Treseder and Save the Children (1997), Shier (2001), Driskell (2017), Smit et al. (2020), and Richards-Schuster and Plachta Elliott (2019) conceptualize levels and degrees of participation from minimal consultation to shared decision-making authority. However, ladder-based and level-oriented models have been critiqued for presenting participation as a procedural and hierarchical progression, often overlooking its contextual, non-linear, and phase-specific nature, as well as the influence of power relations in shaping children’s roles (Teleman et al., 2025). While Lundy et al. (2011) and Imms et al. (2016) identified external and internal factors influencing children’s participation. Together, these frameworks allow for a systematic examination of how participation is operationalized across research phases and power dynamics.

Despite the availability of phase-based and role-based analytical frameworks, reviews of participatory action research with children continue to identify persistent methodological tensions. Research has shown that children’s involvement often varies across stages of the research process, with greater engagement in data collection and more limited influence in agenda-setting, analysis, or dissemination (Jacquez et al., 2013; Lopez-Ordosgoitia et al., 2023; Shamrova & Cummings, 2017). Additionally, recurring challenges have been found, such as institutional research norms, academic hierarchies, and conventional expectations regarding knowledge production, which can constrain the extent to which children exercise meaningful participation within research processes (Bakhtiar et al., 2023).

However, most of these studies focus on outcomes related to Western knowledge rather than Southern perspectives (Hackett, 2019; Hoekstra et al., 2020; Hayward et al., 2021; Simpson & Mendenhall, 2022). This concentration raises a critical question: do similar patterns persist in territories shaped by markedly different structural, historical, and epistemological conditions? Latin America is not simply an “understudied region” within participatory research. It is a site with a historically grounded tradition of action-oriented and dialogical inquiry, articulated through the work of Freire (1970) and Fals-Borda (1987), which foregrounded collective knowledge production and social transformation. This historical trajectory introduces an analytical tension: it remains unclear whether contemporary participatory research with rural children in the region sustains these dialogical and transformative principles, enabling genuine processes of collective knowledge production that challenge existing power relations, or whether it reproduces more instrumental and uneven forms of engagement similar to those documented in predominantly urban Western contexts.

Children and adolescents in rural areas and indigenous communities frequently navigate overlapping dimensions of marginalization linked to territorial inequality, limited institutional presence, economic precarity, and deeply rooted intergenerational authority structures (Houghton et al., 2023). An additional challenge lies in the limited availability of reliable demographic and social data, which restricts the visibility of Indigenous childhoods within research and policy agendas, reinforcing forms of epistemic marginalization that extend beyond material deprivation (FILAC & UNICEF, 2025). These structural conditions influence not only children’s daily lives but also the environments in which research partnerships are formed and sustained.

The importance of examining participation in these contexts is therefore not only geographic but conceptual. Therefore, this review aims to critically analyze participatory research conducted with children and adolescents in rural Latin America between 2018 and 2025, focusing on three central dimensions: (1) the methodological approaches employed; (2) the levels of children’s involvement across distinct research phases; and (3) the sociodemographic characteristics of children. By integrating phase-based frameworks (Israel et al., 2005) with role-based models of participation (Smit et al., 2020) and their sociodemographic characteristics, this study contributes a systematic examination of how participation is operationalized in practice, taking into account the context of the rural children. In doing so, it seeks to clarify whether participatory research in rural Latin America moves beyond inclusion toward shared decision-making, and to identify methodological patterns that may strengthen or constrain meaningful children and adolescent engagement.

## 2. Materials and Methods

This study employs a critical literature review to examine participatory research conducted with children and adolescents in rural Latin America between 2018 and 2025. In this review, “critical” refers to an interpretive and analytical engagement with the literature that goes beyond descriptive synthesis by examining patterns, identifying inconsistencies, and interrogating how children’s participation is constructed across research phases and roles (Ghosh & Choudhury, 2025; Wright & Michailova, 2023).

While the review adopts a structured and transparent search and selection process, it differs from systematic or scoping reviews in its analytical purpose. Rather than aiming to aggregate comparable evidence or map the breadth of the field, this study focuses on critically interpreting a body of participatory research characterized by diverse methodological approaches (Community-Based Participatory Research (CBPR), Participatory Action Research (PAR), and other participatory strategies) and varying conceptualizations of children’s involvement. These features require an analytical approach that can engage with how participation is enacted in practice, rather than treating it as a uniform or comparable outcome.

This approach enables a more in-depth examination of how participation is shaped across contexts, leading to the development of an analytical framework that conceptualizes the redistribution of power across structural, relational, and experiential dimensions.

### 2.1. Selection Strategy and Eligibility Criteria

A structured search was conducted between November 2023 and May 2025. Searches were performed in Scopus, Web of Science, ScienceDirect, and Redalyc to conduct bibliographical research and retrieve pertinent articles written in English and Spanish. Grey literature was explored through Google Scholar and Latin America university repositories. As part of our search strategy, we used the following operators and terms in our title, abstract, and keywords search of the databases, according to DeCS/Mesh: “Community-based Participatory Research” or “Participatory Action Research” or “Youth-led/Child-led Participatory Action Research” and “rural communities” or “rural community” or “rural” and “children” or “youth” and “Latin America”.

To identify potentially overlooked studies, AI-assisted research tools (Elicit, Consensus, and SciSpace) were used to screen related literature and cross-check references. These tools were employed as supplementary aids for article discovery and citation tracking; all inclusion decisions were based on full-text manual review by the authors.

Eligibility criteria were defined to guide the selection of studies, with particular attention to the inclusion of participatory research involving children in Latin America (including countries from both South and Central America). The criteria applied in this review are summarized in Table 1.

**Figure 1:**
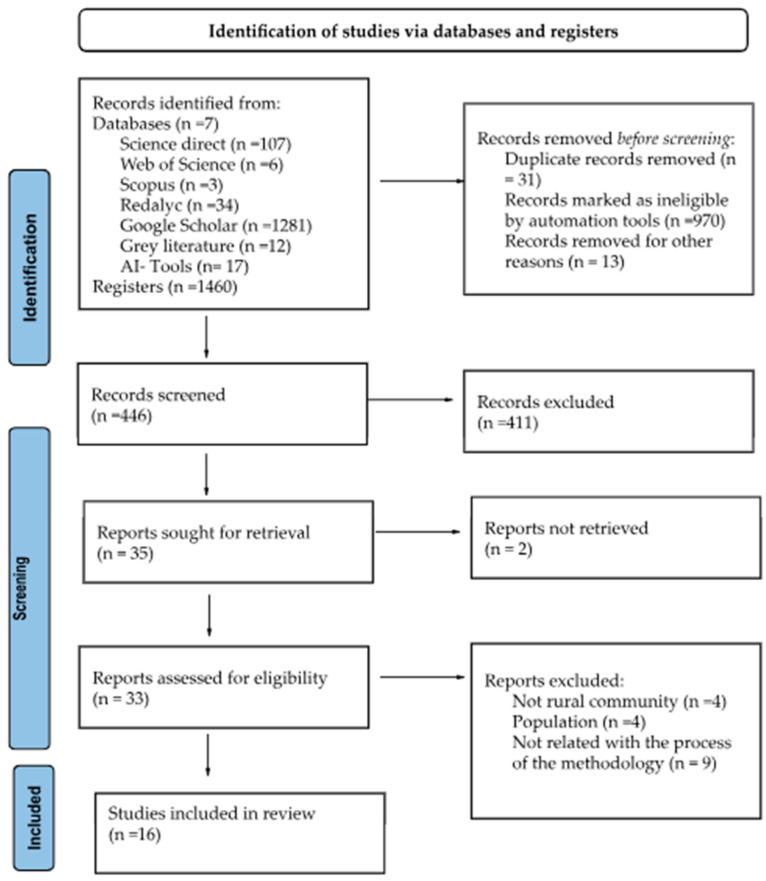
PRISMA study selection process.

Overall, 446 titles and abstract articles from the seven databases were reviewed. After the initial screening, 35 studies were retrieved in full text and examined for inclusion criteria (See Figure 1). Four of these articles were not in rural communities, four were implemented only with adults related to children’s topics, and nine were not related to the methodological process. This process narrowed the sample to 16 relevant studies.

### 2.2. Data Extraction and Analytical Framework

All identified records were imported into Rayyan to facilitate screening and documentation. The first two authors independently evaluated titles, abstracts, and full-text articles for eligibility. Discrepancies were discussed and resolved through consensus to ensure consistency in inclusion decisions. The analysis was structured across three complementary dimensions: the sociodemographic characteristics reported in each study, the phases in which children’s participation occurs (phase-based), and the roles they assume in the research process (role-based).

Sociodemographic characteristics included age, gender, ethnicity, and the research context across studies, and whether these contexts were examined in relation to children’s participation. Participation across research phases was examined following Israel et al. (2005), including: (1) partnership formation and maintenance; (2) co-construction of research topics; (3) research design and implementation; (4) data analysis and interpretation; and (5) dissemination and knowledge translation. To assess the degree of participation by children, we drew primarily on the role-based framework, which classifies participation according to the roles children assume (Smit et al., 2020): (1) none: no participation of the children; (2) inform: information is used without any other type of interaction, including explanations; (3) consult: considering children’s views and suggestions; (4) participate: children participate actively engage in research and knowledge creation, and (5) collaborate: adult and children jointly conduct research and share decision-making. Together, these three dimensions enable a systematic organization of the literature while supporting an analytical examination of how participation is represented across studies.

### 2.3. Use of AI-Assisted Tools

AI-assisted tools were used in a supportive capacity. Platforms such as Elicit, Consensus, and SciSpace were employed to assist in literature exploration and citation cross-checking during the search process. In addition, ChatGPT (OpenAI, GPT-5.3) was used to support language refinement and clarity of academic expression during manuscript preparation. These tools did not participate in study selection, coding, or analytical decision-making. All methodological judgments, coding procedures, and substantive interpretations were conducted exclusively by the authors.

## 3. Results

The final corpus consisted of sixteen empirical studies conducted in rural Latin American contexts (See Table 2). The studies were geographically distributed across Latin America, with representation from Colombia, Mexico, Bolivia, Guatemala, Nicaragua, Ecuador, Honduras, Chile, and Brazil. Thematic areas varied, including health promotion, social sciences, education, agroecology, and territorial governance.

Methodologically, the corpus reflects heterogeneity within participatory research. While some studies were explicitly identified as CBPR, others employed PAR, and citizen science. Despite these variations, all studies claimed to incorporate participatory elements involving children or adolescents.

The analysis of sociodemographic characteristics across the included studies shows variability in how participant populations are described, including dimensions such as age, gender, ethnicity, and structural context. While these characteristics are reported to differing extents, their presence provides a basis for considering how multiple social dimensions may shape participatory processes. However, across the studies, these characteristics are predominantly presented in isolation, and their analytical integration remains limited (Table 3).

Regarding age groups, five studies focused primarily on younger children (under 12 years) (Canul et al., 2022; Bayona Castañeda et al., 2020; Jardim et al., 2023; Lay-Lisboa et al., 2018; Padilla & Vélez, 2022), whereas the majority centered on adolescents. Three studies included broader youth ranges extending into early adulthood (Ciborowski et al., 2022; Peña-Ochoa & Bonhomme, 2018; Sarigumba et al., 2023), and in three cases, participants were described more generally as “children and adolescents” without precise age specification (Arenas-Monreal et al., 2024; Dodd et al., 2024; Elao Bonifaz, 2018).

In relation to gender, seven of the sixteen studies explicitly reported the number of male and female participants (Canul et al., 2022; Bayona Castañeda et al., 2020; Gallego et al., 2023; Montes et al., 2022; Peña-Torres & Reina-Rozo, 2022; Sarigumba et al., 2023; Uscanga et al., 2019). Among these, five studies reported a higher proportion of female participants, suggesting a greater representation of girls and young women within the samples. In the remaining studies, gender was either mentioned without disaggregation or not specified.

With respect to ethnicity, only four studies out of the 16 explicitly engaged with Indigenous populations (Canul et al., 2022; Ciborowski et al., 2022; Jardim et al., 2023; Sarigumba et al., 2023). In the remaining studies, ethnicity was not specified, limiting the available information on how participant backgrounds were reported across the literature.

Regarding research context, reported conditions included poverty, low levels of education, social exclusion, migration, and limited access to basic services. Only two of the sixteen studies showed a partial consideration of multiple social dimensions of the rural communities within their analysis (Ciborowski et al., 2022; Peña-Ochoa & Bonhomme, 2018), while in another, gender was considered as a dimension of differentiation (Sarigumba et al., 2023). In contrast, twelve studies described the characteristics of the communities in primarily descriptive terms, without integrating these dimensions into the analysis of participation; one study did not report this information (Peña-Torres & Reina-Rozo, 2022).

### Distribution of Participation Across Research Phases

The analysis examined children’s involvement across five research phases: (1) partnership formation and maintenance; (2) co-construction of research topics; (3) research design and implementation; (4) data analysis and interpretation; and (5) dissemination and knowledge translation. Participation was assessed using both phase-based and role-based frameworks.

Children’s involvement in the first phase, partnership formation, and maintenance was relatively limited. Most studies either did not mention the involvement of children (Padilla & Vélez, 2022; Peña-Ochoa & Bonhomme, 2018; Peña-Torres & Reina-Rozo, 2022) or they took children at the “inform” level (Arenas-Monreal et al., 2024; Canul et al., 2022; Gallego et al., 2023; Jardim et al., 2023; Lay-Lisboa et al., 2018; Uscanga et al., 2019). For instance, in Gallego et al. (2023), participation was operationalized through a unidirectional information process: a science teacher introduced the project in the classroom, invited students to participate, and provided them with information sheets to be taken home to their parents. In three of the 16 articles, children did not participate in this phase, with partnerships established primarily with community leaders, teachers, and parents (Ciborowski et al., 2022; Montes et al., 2022). Only three studies reported active child involvement in building relationships with researchers (Bayona Castañeda et al., 2020; Dodd et al., 2024; Sarigumba et al., 2023). These interactions allowed for the integration of children’s perspectives regarding their community and social environment into the early stages of the project.

During phase two, co-constructing joint topics, children’s engagement increases. Seven of the 16 studies connected community needs with the research project, ensuring that children were consulted about the topics of interest (Amorocho et al., 2024; Canul et al., 2022; Jardim et al., 2023; Lay-Lisboa et al., 2018; Montes et al., 2022; Peña-Ochoa & Bonhomme, 2018; Uscanga et al., 2019). In this sense, Jardim et al. (2023) held targeted meetings where students, alongside other stakeholders, voiced their specific concerns regarding health priorities and the urgent need for health promotion programs in their territory. In contrast, three articles involved the children in validating the research themes (Bayona Castañeda et al., 2020; Dodd et al., 2024; Sarigumba et al., 2023), with one forming an Advisory Youth Committee for consultations (Peña-Torres & Reina-Rozo, 2022). However, the remaining articles either did not mention the co-construction of the joint topics (Gallego et al., 2023; Padilla & Vélez, 2022) or conducted this phase with adults only (Ciborowski et al., 2022; Elao Bonifaz, 2018; Arenas-Monreal et al., 2024).

During phase three, involving the children in designing and conducting the research, children’s participation varied. Nevertheless, in this phase, there was an incrementation in the level of children’s participation in decision-making, especially in three studies (Bayona Castañeda et al., 2020; Dodd et al., 2024; Sarigumba et al., 2023). For instance, Sarigumba et al. (2023) described a process in which children assumed active roles in defining research priorities through visioning workshops, allowing them to participate in decision-making regarding the activities and themes that shaped the study’s trajectory. Five of the articles specifically highlighted the children’s involvement in data collection, but there was less emphasis on their participation in designing the methodology (Ciborowski et al., 2022; Gallego et al., 2023; Jardim et al., 2023; Peña-Torres & Reina-Rozo, 2022; Uscanga et al., 2019). In Uscanga et al., participation was operationalized through structured tasks in which students took photographs of community resources and responded to a predefined prompt about their relevance. In two of the sixteen articles, the authors already had a design and a topic, so they only consulted the children to validate it without them having an active involvement on it (Arenas-Monreal et al., 2024; Elao Bonifaz, 2018; Padilla & Vélez, 2022). The remaining articles did not mention the procedure for this phase (Canul et al., 2022; Montes et al., 2022; Peña-Ochoa & Bonhomme, 2018).

In phase four, participation in data analysis and interpreting results, five articles mention a consulting process with the children about the analysis made by the researchers (Amorocho et al., 2024; Arenas-Monreal et al., 2024; Ciborowski et al., 2022; Gallego et al., 2023; Jardim et al., 2023; Montes et al., 2022; Peña-Torres & Reina-Rozo, 2022; Uscanga et al., 2019). For example, in Ciborowski et al. (2022), preliminary themes identified by researchers were shared with participants and community leaders for validation. In some cases, researchers prepared the topics for analysis and presented them to the children and the community in discussion groups. In other cases, especially when using photovoice techniques, children analyzed their photos with the researchers. Only three articles mentioned how the children took ownership of the analysis process as active co-researchers (Bayona Castañeda et al., 2020; Dodd et al., 2024; Sarigumba et al., 2023). The four remaining articles did not mention this phase (Canul et al., 2022; Elao Bonifaz, 2018; Lay-Lisboa et al., 2018; Padilla & Vélez, 2022).

In the fifth phase, dissemination and presentation of the results, children were involved at different levels of engagement. Seven articles mentioned the process of result dissemination through oral and written expression done by the children. This included discussions with the stakeholders and the community leaders that resulted in agreements on relevant actions (Amorocho et al., 2024; Arenas-Monreal et al., 2024; Ciborowski et al., 2022; Elao Bonifaz, 2018; Gallego et al., 2023; Montes et al., 2022; Peña-Torres & Reina-Rozo, 2022). Peña-Torres and Reina-Rozo (2022) demonstrated this engagement through the ‘Periódico del Futuro’ (Newspaper of the Future), a tool designed for participants to explore and create future scenarios for their territory, materializing their proposals through both oral and written expression. On the other hand, two articles mentioned how children focused on using the results to create social change. In these articles, the discussion and the presentation of the results were done throughout all the CBPR phases and not just at the end of the research (Bayona Castañeda et al., 2020; Sarigumba et al., 2023). The other five articles do not mention this phase (Canul et al., 2022; Dodd et al., 2024; Lay-Lisboa et al., 2018; Padilla & Vélez, 2022; Peña-Ochoa & Bonhomme, 2018; Uscanga et al., 2019). Among these articles, one involved children in producing a theater presentation on health issues. However, there is insufficient information in the previous phases to assess the level of children’s participation in this phase (Padilla & Vélez, 2022).

**Figure 2:**
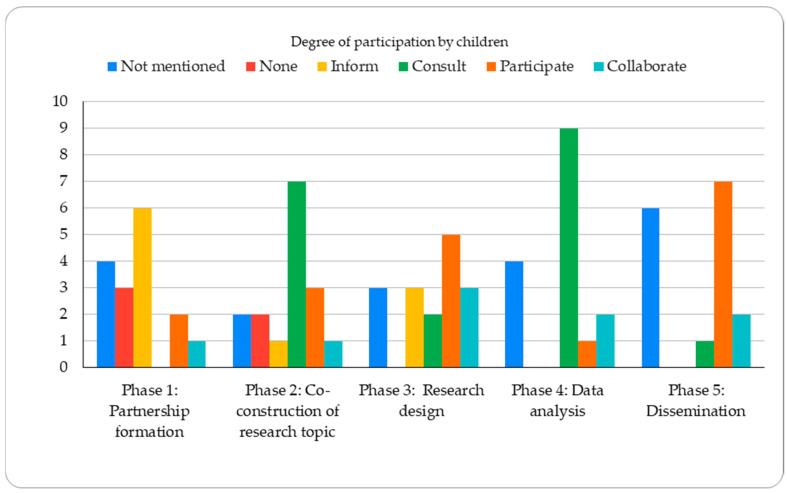
Degree of children’s participation per phase, offering an overall view of the most frequently employed levels.

As shown in Figure 2, children predominantly served as recipients in phase 1. In phase 2, in approximately half of the studies, the children were consulted, while only in four of them they participated or co-constructed the joint topics. In phase 3, children predominantly assumed active roles in conducting the research. In phase 4, most children were consulted during the analysis, and in phase 5, most studies involved children as active participants.

## 4. Discussion

Participation in participatory research with children in rural Latin America, as evidenced across the reviewed studies, is fundamentally structured by the extent to which power is redistributed across the research process, rather than by the mere adoption of participatory methods. The findings suggest that this redistribution operates across three interrelated levels: (1) a structural–methodological level; (2) a relational–community level; and (3) an experiential–child level. These levels are mutually constitutive, such that meaningful participation of children depends on how power is configured across structural and relational levels. Participation, therefore, cannot be reduced to involvement in discrete activities, but must be understood as a multi-level process through which authority is either redistributed or reproduced, ultimately shaping whether participation becomes meaningful or remains procedural.

At the structural–methodological level, the shared decision-making remains uneven and often constrained from the outset of the research process. While methodological flexibility, facilitated through approaches such as photovoice, focus groups, theater, or art-based strategies, often enhanced children’s engagement during implementation and dissemination, early-phase agenda-setting and analysis frequently remained adult-led. This suggests that methodological creativity, in itself, does not constitute a redistribution of power, but may instead operate within pre-existing structures that delimit the scope of children’s influence. As Cory et al. (2024) argue that meaningful participation requires an explicit shift in power rather than mere methodological innovation. This are not merely methodological but structural, manifesting in two key dimensions: institutional and territorial pre-structuring, and the invisibility of sociodemographic interactions.

First, the institutional and territorial pre-structuring are the conditions under which research is funded, designed, and implemented. Institutional requirements, constrained timelines, and logistical challenges associated with rural contexts shape what forms of participation are considered feasible, often privileging efficiency over inclusivity. Within this configuration, participation is already selectively structured, excluding certain groups of children before the research even begins. The review highlights, for instance, the limited inclusion of Indigenous children, younger children (particularly those under 12), and, in some cases, boys, whose participation is less frequently facilitated or documented. In this sense, exclusion operates at the level of research design itself, where decisions about whom to engage actively shape whose voices are included and whose are rendered absent.

This initial selectivity leads directly to the second dimension: the invisibility of sociodemographic interactions. Importantly, none of the reviewed studies explicitly analyzed the interaction among sociodemographic characteristics when examining children’s participation in rural contexts. This analytical fragmentation reinforces a tendency to approach “rural children” as a relatively uniform category, overlooking how participation is actually shaped through the interplay of multiple social positions. Rather than claiming an intersectional analysis, this review identifies a critical gap in the literature: the need for research frameworks capable of capturing the multidimensionality of rural childhoods. This has important implications, as it risks overlooking how participation is shaped through the interplay of gender, age, ethnicity, and structural inequalities (Houghton et al., 2023). Without engaging with how these dimensions intersect, participatory research may inadequately capture how power operates across children’s everyday lives and how participation is differentially enabled or constrained (Konstantoni & Emejulu, 2017; Miranda, 2018).

At the relational–community level, the intergenerational negotiation of authority and ownership emerges as a defining feature of participatory research in rural contexts. Participation unfolded not only between researchers and children but also within community hierarchies shaped by adult authority, cultural norms, and institutional actors. In rural territories, where community governance structures are often deeply embedded, researchers navigate dual power dynamics: negotiating authority with children while simultaneously engaging adult community leadership (Mazó, 2025).

This dual negotiation reveals that children’s participation is not directly accessible, but socially mediated, which helps explain their limited involvement in key phases such as partnership formation and analytical interpretation. However, rather than critically interrogating these dynamics, some tend to treat community authority as a contextual backdrop rather than as an active site of power that shapes the boundaries of participation. Mainstream participation frameworks tend to focus on direct researcher–child dyads, overlooking how access to children’s voices is filtered through intergenerational relations and culturally embedded systems of governance (Bradbury-Jones et al., 2018).

Moving toward meaningful participation therefore requires shifting from binary frameworks that oppose adult control to child autonomy. This entails not only recognizing adult authority, but making it visible as a site of negotiation, where roles, responsibilities, and decision-making power are explicitly discussed and reconfigured from the outset of the research process.

At the experiential–child level, the differentiated roles children assume across research phases shape how participation is lived and experienced by children themselves. In this sense, the role a child inhabits, whether as an informed participant, collaborator, or co-researcher, does not inherently determine the meaningfulness of their participation. Instead, it reflects the boundaries of agency made available within the research design and its broader relational context. Where structural and intergenerational redistribution of power is limited, children’s participation tends to be concentrated in implementation and dissemination phases, with restricted influence over agenda-setting or knowledge production.

Building on this, and following Smit et al. (2020), these shifting roles can be understood as expressions of how agency is structured and enacted across different moments of the research process. However, rather than representing purely methodological variations, these roles indicate the extent to which children are able to meaningfully engage, contribute, and make sense of their involvement within the research process (Kellett, 2010). In this sense, agency is not only expressed through visible involvement, but also through forms of negotiation, selective engagement, resistance, or silence (Atkinson, 2019) Participation is not a static methodological requirement, it is a dynamic and situated experience that unfolds across interactions, relationships, and moments of involvement (Fielding, 2011). From this perspective, participation must be understood in relation to children’s processes of meaning-making.

Taken together, the three levels identified in this review suggest that meaningful participation emerges not from isolated methodological choices or children’s involvement in discrete research phases, but from how power is redistributed across structural, relational, and experiential levels. It is through the alignment of these levels that participation moves beyond inclusion, enabling children to engage with and shape their involvement in ways that are meaningful to them. When this redistribution is partial or unequal, participation tends to remain procedural (Hart, 1992; Treseder & Save the Children, 1997; Shier, 2001); when more consistently enacted, it creates the conditions for deeper forms of engagement grounded in children’s lived experiences (Mensa-Kwao et al., 2024).

In this sense, this review shows that meaningful participation lies not only in the redistribution of power, but in the extent to which this redistribution enables children to recognize themselves as subjects of the research process, rather than merely as participants within it (Brown, 2022; Cory et al., 2024).

## 5. Conclusions

This critical review examined participatory research with children and adolescents in rural Latin America between 2018 and 2025, focusing on sociodemographic characteristics, research phases, and the degree of children’s participation. The findings indicate that, while participatory approaches are widely adopted, children’s participation remains uneven across research phases and contexts. Importantly, this review shows that participation cannot be fully understood through methodological approaches or levels of involvement alone, but must be examined in relation to how power is distributed and negotiated throughout the research process. In this sense, meaningful participation depends on the extent to which structural conditions, intergenerational dynamics, and children’s lived experiences are addressed in an integrated way. When these dimensions are only partially considered, participation may remain limited in its capacity to enable children to influence and make sense of their involvement in research. Ultimately, fostering meaningful participation entails a fundamental shift in the researcher’s role: from managing children’s voices to enabling the structural and relational conditions where children can recognize themselves as sovereign subjects of knowledge production within their own territories.

This review should be interpreted in light of some limitations. First, the conceptualization of “rurality” in Latin America is complex and context-dependent, which may have influenced the identification and categorization of studies. Second, the exclusion of Portuguese-language databases may have limited the inclusion of research from Brazil. Third, although sociodemographic characteristics were reported in most studies, these were often presented descriptively, which constrained the analysis of how intersecting factors shape participation. While these limitations do not undermine the overall findings, they highlight areas for further refinement in future research and review processes. To advance the field, future research must move beyond the descriptive reporting of participation toward integrated analytical frameworks that address the invisibility of sociodemographic interactions.

## Figures and Tables

**Table 1:** Eligibility criteria applied in the scoping review.

Domain	Criteria
Study type	Empirical studies (qualitative, quantitative, or mixed methods
Geographic scope	Rural communities of Latin America
Population	Children and/or adolescents (0–17 years)
Approach	Participatory research approaches
Participation requirement	Evidence of children’s active participation in at least one research phase
Language	Spanish/English

**Table 2:** Summary characteristics of selected studies, and involvement of children in the five phases of CBPR.

Authors	Approach	Area of Study	Country	Research Techniques
Peña-Torres and Reina-Rozo (2022)	Citizen Science	Agroecology	Colombia	Creation of an Advisory Youth Group Learning by doing
Canul et al. (2022)	CBPR	Health	Mexico	Interviews Focus group Health intervention
Bayona Castañeda et al. (2020)	PAR	Social Sciences	Colombia	Social Cartography Urban transects and narratives of place Interviews and focus groups Ranking techniques Workshops on proposal elaboration
Montes et al. (2022)	Citizen Science	Health and Well-being	Colombia	Technology-enabled data collection by photovoice Participatory mapping workshops Participatory community meeting
Padilla and Vélez (2022)	CBPR	Health	Mexico	Puppet theater
Peña-Ochoa and Bonhomme (2018)	PAR	Education	Chile	Social identity game Learning objects Subjective maps of the school Group discussions Workshops
Ciborowski et al. (2022)	CBPR	Health	Guatemala	Focus Groups Photovoice
Elao Bonifaz (2018)	CBPR	Social Sciences	Ecuador	Semi-structured interviews Non-participant observation Informal conversations
Sarigumba et al. (2023)	PAR	Territorial governance	Bolivia	Visioning workshop focused on the voice of youth Focus groups and follow-up workshop In-depth research working with individual youth A large-scale gathering of youth
Gallego et al. (2023)	PAR	Social Sciences	Bolivia	Focus Groups Photovoice
Uscanga et al. (2019)	PAR	Entrepreneurship	Nicaragua	Photovoice
Arenas-Monreal et al. (2024)	CBPR	Health	Mexico	Mixed methods, diagnostics, and community initiative
Lay-Lisboa et al. (2018)	PAR	Education	Chile	Mind maps Matrices of present and future scenarios
Amorocho et al. (2024)	PAR	Education	Colombia	Not specified
Dodd et al. (2024)	PAR	Agroecology	Honduras	Not specified
Jardim et al. (2023)	PAR	Health	Brazil	Mixed methods, concept mapping

**Table 3:** Sociodemographic characteristics of the children.

Nº	Authors	Sociodemographic Characteristics				
		Age	Gender F: Female M: Male	Ethnicity/Indigenous Identity	Research Context	Analysis of the Sociodemographic Characteristics
1	Peña-Torres and Reina-Rozo (2022)	14–22 years	Yes (62.5% F, 37.5% M)	Not specified (peasant identity implied)	Not specified	No
2	Canul et al. (2022)	5–14 years	Yes (51.5% M, 48.5% F)	Yes (community of Maya origin)	Yes (housing, employment, access to services described)	No, descriptive sample only.
3	Bayona Castañeda et al. (2020)	7–17 years	Yes (36% M, 64% F)	Not specified	Yes (low-income, overcrowding, high density, environmental risk)	No, descriptive and contextual only.
4	Montes et al. (2022)	13–17 years	Yes (72.7% F, 27.3% M)	No (Afro-descendant population)	Yes (multidimensional poverty, precarious work, poor housing, lack of services, violence, limited state presence)	No, descriptive and contextual only.
5	Padilla and Vélez (2022)	8–10 years	Not specified	Not specified	Yes (schools located in areas with medium and high marginalization; access to internet and digital tools described)	No, descriptive and contextual only.
6	Peña-Ochoa and Bonhomme (2018)	13–14 years	Yes (mixed groups; boys and girls)	Not specified	Yes (high-vulnerability schools; peripheral urban and rural contexts; high poverty and school dropout rates; socio-spatial segregation)	Partial, social categories recognized theoretically and structurally.
7	Ciborowski et al. (2022)	16–65 years	Yes (boys and girls included)	Yes (Indigenous identity explicitly present)	Yes (rural/remote Indigenous communities; structural inequities, colonial context, limited access to services)	Partial, implicit analysis of characteristics of the community.
8	Elao Bonifaz (2018)	Adolescents	Not specified	Not specified	Yes (context of social vulnerability; marginalization; civic participation systems in unequal settings	No, descriptive and contextual only.
9	Sarigumba et al. (2023)	15–26 years	Yes (46.4% F, 53.6% M)	Yes (Indigenous youth from specific communities)	Yes (Indigenous rural territories; dependence on natural resources; environmental degradation; limited livelihood opportunities; marginalization and governance challenges)	Partial, identity and gender roles discussed.
10	Gallego et al. (2023)	12–17 years	Yes (80% F, 20 M)	Not explicitly specified (Indigenous heritage implied; Aymara language context; strong cultural references)	Yes (rural municipality; high poverty, inequality, limited internet access, low infrastructure, informal economy, political crisis context)	No, descriptive and contextual only.
11	Uscanga et al. (2019)	16 years	Yes (75% F, 25% M)	Not specified	Yes (low-income rural youth; average family size of six; resource-constrained context; limited access to technology such as mobile phones)	No, descriptive and contextual only.
12	Arenas-Monreal et al. (2024)	Children and Adolescent	Yes (the number is not specified)	Not specified	Yes (High migration rate, poverty context)	No, descriptive and contextual only.
13	Lay-Lisboa et al. (2018)	10–11 years	Yes (the number is not specified)	Not specified	Yes (public school in socially vulnerable context; references to inequality, exclusion, and school as a space of social reproduction and citizenship formation)	No, descriptive and contextual only.
14	Amorocho et al. (2024)	15–17 years	Gender not specified for children/youth participants	Not specified	Yes (multidimensional poverty; illiteracy; low educational achievement; rural–urban inequality; limited access to water, gas, internet; geographic isolation; conflict and state neglect)	No, descriptive and contextual only.
15	Dodd et al. (2024)	Youth	Partial (mentions inclusion of women and gender norms, but no sample breakdown)	Not specified	Limited (rural setting mentioned; no detailed socio-economic or structural data)	No, descriptive and contextual only.
16	Jardim et al. (2023)	9–17 years	Yes (the number is not specified)	Yes (Indigenous youth explicitly stated as target population)	Limited (Indigenous context acknowledged; no detailed socio-economic or living condition data)	No, descriptive and contextual only.

## Data Availability

Data is contained within the article.
